# Supplementary material for: The CAGE complex: a hollow, megadalton, protein assembly in prokaryotic and eukaryotic microbes
Source: bioRxiv. 2025 Sep 22:2025.09.22.677704. Preprint. [Version 1] doi: 10.1101/2025.09.22.677704 (PMC12485934; doi:10.1101/2025.09.22.677704)
Supplement: Supplement 1 — Figure S1 - Cryo-EM workflow and statistics for Tetrahymena CAGE1 complex Figure S2 - Cryo-EM workflow and statistics for Dictyostelium CAGE complex [file media-1.pdf]

Supplemental Figure Legends

Figures S1-S2. Cryo-EM workflow and statistics for (S1) *Tetrahymena* CAGE1 complex and (S2) *Dictyostelium* CAGE complex. In each case, the specific cryo-EM workflow is presented with a view of the final EM density along with the statistics of the final reconstruction. (A) Data processing and refinement pipeline for the complex using cryoSPARC v4 (*Tetrahymena*) and v4.5 (*Dictyostelium*). The symmetry applied at each refinement step is indicated at the lower right of the respective refinement. (B) Cryo-EM volume colored by local resolution estimation (units in Å). (C) Gold-standard Fourier shell correlation (GSFSC) curves calculated in cryoSPARC, with the 0.143 threshold. (D) Viewing angle distribution plot.

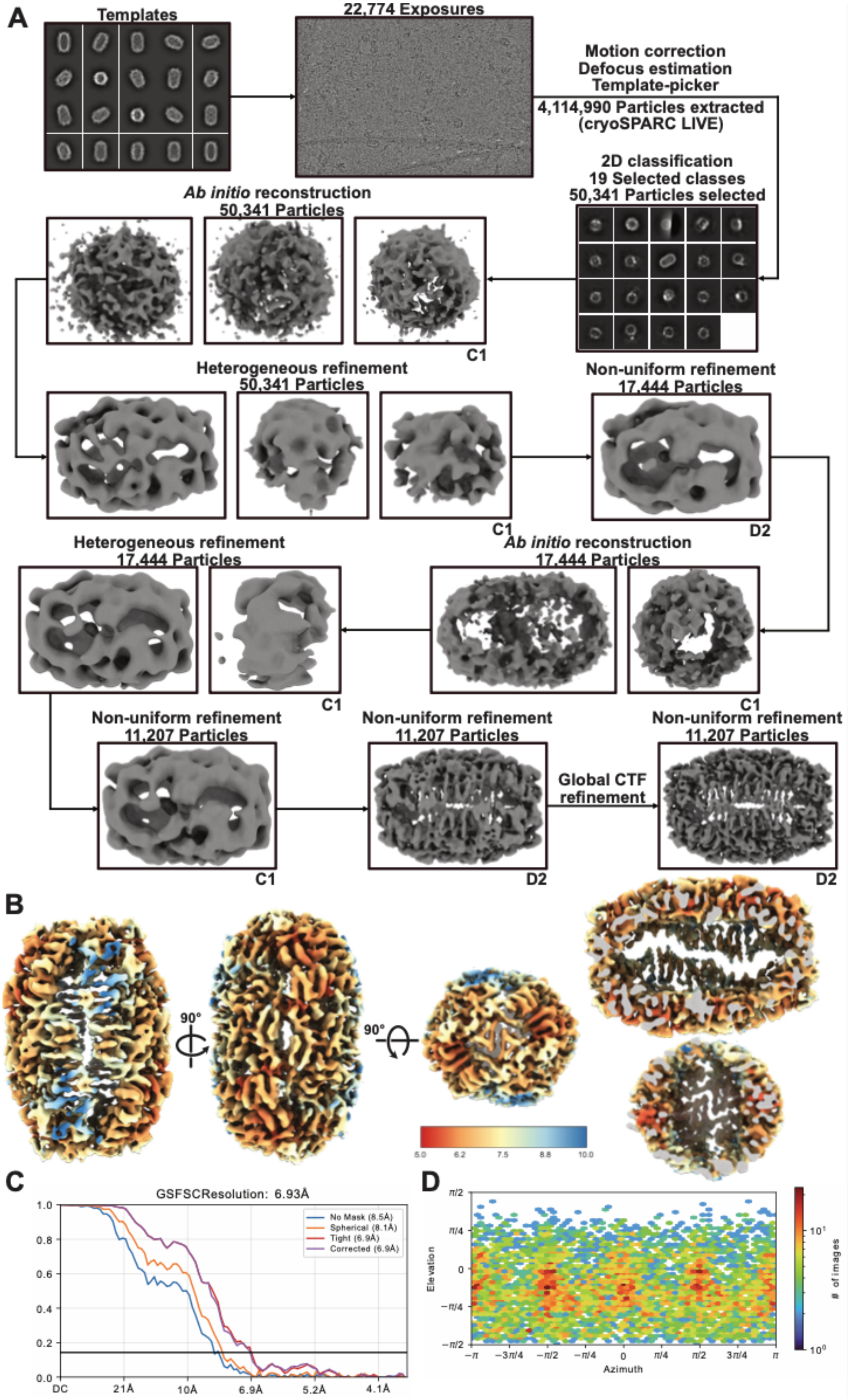

Figures S1. Cryo-EM workflow and statistics for *Tetrahymena* CAGE1 complex.

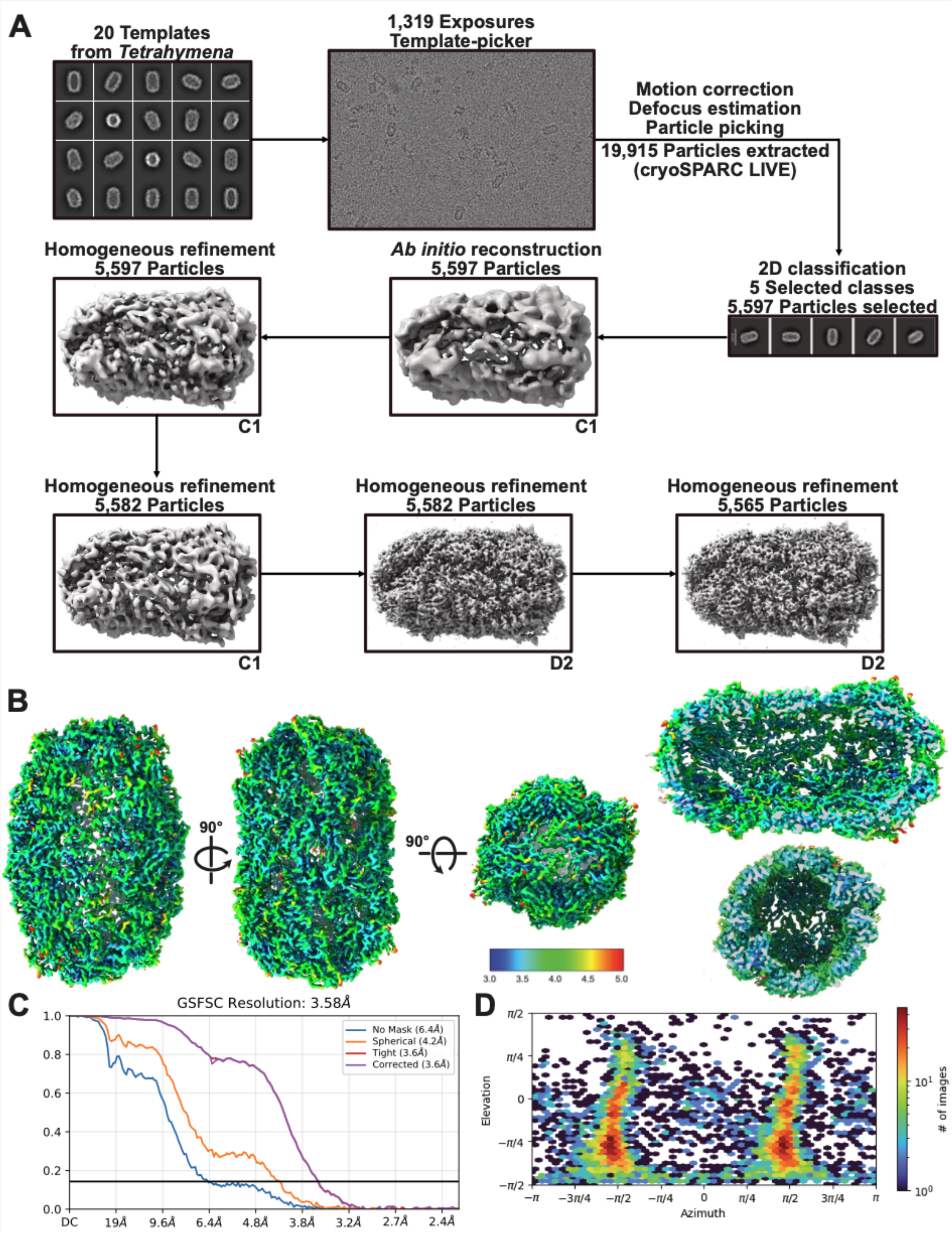

Figures S2. Cryo-EM workflow and statistics for *Dictyostelium* CAGE complex.
