# Supplementary material for: The CAGE complex: a hollow, megadalton, protein assembly in prokaryotic and eukaryotic microbes
Source: bioRxiv. 2025 Sep 22:2025.09.22.677704. Preprint. [Version 1] doi: 10.1101/2025.09.22.677704 (PMC12485934; doi:10.1101/2025.09.22.677704)
Supplement: Supplement 2 — File S1 - Interactive Krona plot.html [file media-2.html]

xml version="1.0" encoding="UTF-8"?

Javascript must be enabled to view this page.

magnitude
score


CAGE complex


 3521

 1


 638

 1


 14

 1


 10

 1


 8

 1


 8

 1


 5

 1


 4

 1


 3

 1


 1

 1


 1

 1


 1

 1


 3

 1


 2

 1


 1

 1


 1

 1


 1

 1


 1

 1


 2

 1


 2

 1


 2

 1


 2

 1


 4

 1


 4

 1


 4

 1


 4

 1


 4

 1


 316

 1


 316

 1


 286

 1


 188

 1


 188

 1


 188

 1


 188

 1


 53

 1


 2

 1


 35

 1


 5

 1


 12

 1


 4

 1


 36

 1


 11

 1


 5

 1


 17

 1


 4

 1


 2

 1


 2

 1


 98

 1


 7

 1


 7

 1


 7

 1


 7

 1


 83

 1


 83

 1


 1

 1


 1

 1


 1

 1


 1

 1


 56

 1


 22

 1


 8

 1


 3

 1


 1

 1


 4

 1


 2

 1


 2

 1


 1

 1


 1

 1


 27

 1


 26

 1


 1

 1


 4

 1


 3

 1


 2

 1


 1

 1


 1

 1


 1

 1


 1

 1


 1

 1


 1

 1


 1

 1


 1

 1


 1

 1


 7

 1


 3

 1


 1

 1


 1

 1


 1

 1


 1

 1


 12

 1


 5

 1


 1

 1


 1

 1


 1

 1


 2

 1


 1

 1


 1

 1


 6

 1


 5

 1


 1

 1


 8

 1


 8

 1


 8

 1


 3

 1


 5

 1


 28

 1


 28

 1


 7

 1


 7

 1


 7

 1


 2

 1


 3

 1


 2

 1


 20

 1


 3

 1


 2

 1


 2

 1


 1

 1


 1

 1


 1

 1


 1

 1


 1

 1


 16

 1


 16

 1


 3

 1


 7

 1


 2

 1


 1

 1


 1

 1


 2

 1


 1

 1


 1

 1


 1

 1


 1

 1


 2

 1


 2

 1


 2

 1


 2

 1


 2

 1


 2

 1


 220

 1


 133

 1


 27

 1


 27

 1


 8

 1


 8

 1


 8

 1


 19

 1


 19

 1


 19

 1


 104

 1


 37

 1


 37

 1


 30

 1


 2

 1


 3

 1


 2

 1


 14

 1


 14

 1


 7

 1


 7

 1


 52

 1


 52

 1


 52

 1


 1

 1


 1

 1


 2

 1


 2

 1


 2

 1


 1

 1


 1

 1


 1

 1


 1

 1


 1

 1


 1

 1


 69

 1


 5

 1


 5

 1


 2

 1


 2

 1


 3

 1


 3

 1


 64

 1


 53

 1


 45

 1


 10

 1


 9

 1


 9

 1


 9

 1


 1

 1


 1

 1


 30

 1


 30

 1


 30

 1


 5

 1


 5

 1


 5

 1


 5

 1


 8

 1


 3

 1


 3

 1


 3

 1


 3

 1


 5

 1


 5

 1


 3

 1


 3

 1


 3

 1


 2

 1


 2

 1


 11

 1


 11

 1


 11

 1


 6

 1


 6

 1


 5

 1


 5

 1


 18

 1


 18

 1


 18

 1


 18

 1


 18

 1


 18

 1


 45

 1


 32

 1


 28

 1


 11

 1


 9

 1


 9

 1


 8

 1


 8

 1


 4

 1


 4

 1


 8

 1


 3

 1


 1

 1


 4

 1


 5

 1


 5

 1


 1

 1


 1

 1


 1

 1


 1

 1


 1

 1


 1

 1


 9

 1


 9

 1


 9

 1


 9

 1


 9

 1


 9

 1


 1

 1


 8

 1


 7

 1


 1

 1


 22

 1


 22

 1


 3

 1


 3

 1


 3

 1


 3

 1


 19

 1


 1

 1


 1

 1


 1

 1


 1

 1


 1

 1


 1

 1


 18

 1


 18

 1


 14

 1


 14

 1


 14

 1


 4

 1


 1

 1


 1

 1


 3

 1


 3

 1


 1

 1


 1

 1


 1

 1


 1

 1


 1

 1


 1

 1


 1

 1


 1

 1


 1

 1


 1

 1


 1

 1


 1

 1


 2878

 1


 2734

 1


 1424

 1


 693

 1


 342

 1


 288

 1


 7

 1


 1

 1


 20

 1


 3

 1


 4

 1


 14

 1


 9

 1


 23

 1


 45

 1


 4

 1


 5

 1


 8

 1


 4

 1


 4

 1


 1

 1


 3

 1


 3

 1


 6

 1


 92

 1


 78

 1


 8

 1


 3

 1


 2

 1


 228

 1


 185

 1


 11

 1


 7

 1


 1

 1


 1

 1


 2

 1


 2

 1


 1

 1


 3

 1


 1

 1


 2

 1


 52

 1


 17

 1


 8

 1


 6

 1


 2

 1


 2

 1


 2

 1


 4

 1


 4

 1


 4

 1


 4

 1


 27

 1


 55

 1


 36

 1


 36

 1


 18

 1


 4

 1


 3

 1


 13

 1


 2

 1


 15

 1


 15

 1


 15

 1


 15

 1


 835

 1


 74

 1


 22

 1


 1

 1


 1

 1


 478

 1


 455

 1


 154

 1


 52

 1


 9

 1


 11

 1


 27

 1


 6

 1


 1

 1


 19

 1


 1

 1


 194

 1


 4

 1


 4

 1


 6

 1


 3

 1


 3

 1


 55

 1


 55

 1


 54

 1


 19

 1


 101

 1


 92

 1


 209

 1


 58

 1


 35

 1


 1

 1


 1

 1


 132

 1


 28

 1


 28

 1


 14

 1


 10

 1


 1

 1


 17

 1


 4

 1


 4

 1


 4

 1


 7

 1


 4

 1


 87

 1


 87

 1


 2

 1


 36

 1


 22

 1


 3

 1


 2

 1


 11

 1


 30

 1


 12

 1


 3

 1


 5

 1


 2

 1


 1

 1


 11

 1


 1

 1


 1

 1


 1

 1


 1

 1


 5

 1


 4

 1


 2

 1


 2

 1


 1

 1


 3

 1


 3

 1


 1

 1


 1

 1


 1

 1


 1

 1


 4

 1


 1

 1


 1

 1


 1

 1


 6

 1


 2

 1


 1

 1


 1

 1


 1

 1


 3

 1


 3

 1


 17

 1


 6

 1


 1

 1


 1

 1


 1

 1


 3

 1


 2

 1


 2

 1


 1

 1


 1

 1


 5

 1


 2

 1


 2

 1


 1

 1


 1

 1


 1

 1


 1

 1


 1

 1


 1

 1


 1

 1


 5

 1


 3

 1


 1

 1


 2

 1


 2

 1


 2

 1


 2

 1


 2

 1


 1

 1


 1

 1


 1

 1


 1

 1


 1

 1


 25

 1


 9

 1


 9

 1


 9

 1


 9

 1


 9

 1


 1

 1


 3

 1


 5

 1


 1

 1


 1

 1


 1

 1


 1

 1


 1

 1


 1

 1


 1

 1


 5

 1


 2

 1


 2

 1
